# Using the HEART score in patients with chest pain in the emergency department of Kosovo as an important triage criterion for treatment and hospitalization

**DOI:** 10.1097/MS9.0000000000000940

**Published:** 2023-06-08

**Authors:** Premtim Rashiti, Albiona Rashiti – Bytyçi, Kadri Haradinaj, Erolind Dermaku, Valdet Berisha, Valon Bellanica, Leonora Svarça, Vjollca Haklaj, Vendenis Lahu, Egzon Duraku, Afrim Shabani

**Affiliations:** a Medical Faculty; b Department of Epidemiology, National Institute of Public Health, University of Prishtina “Hasan Prishtina”, Prishtina, Kosovo; c University Clinical Center of Kosovo, Hospital Circle Pristina, Kosovo

**Keywords:** Chest pain, emergency department, troponin

## Abstract

Despite being a frequent presenting complaint in emergency rooms, chest pain’s clinical therapy varies greatly. Our objectives were to identify the traits of people who complain of chest pain and to analyze the value of the HEART (history, electrocardiogram, age, risk factors, and initial troponin) score index for risk assessment. Depending on how bad it is, each abnormality can be given a score of zero, one, or two points. These five factors add up to the HEART score.

**Methods:**

Clinical information on 269 individuals with chest pain admitted to the Emergency Room was reviewed: January 2022 until January 2023. A prospective registry was used to record information about patients with nontraumatic chest discomfort who were admitted from the emergency department.

**Results:**

Over a 12-month period, patients admitted in emergency department were classified through HEART score. From them 101 patients (37%) belong to the age group older than or equal to 65 years, 134 patients (50%) belong to the age group 45–65 years, and 34 patients (13%) belong to the age group younger than or equal to 45 years. Strong positive correlation between troponin level (HEART score) and hospitalization, also *p* value 0.043 is typically considered to be statistically significant. According to HEART score classification 43 cases (60%) from the group with 7–10 (high risk) were hospitalized. According to anamnesis (history) on cardiovascular disease in relation to hospitalization, then 48 cases hospitalized (67%) belong to the classification 1-moderately suspicious and 21 cases hospitalized (29%) belong to the classification 2-highly suspicious classification.

**Conclusion:**

The HEART score can be used for triage since it is a simple, rapid, and accurate predictor of outcome in patients with chest pain. A medium risk group included about half of the patients who reported chest pain to an emergency room. Hospitalization and troponin level had a strong positive link (HEART score), with a p value of 0.043.

## Introduction

Highlights

- Identify the traits of people who complain of chest pain and to analyze the value of the HEART.
- Two hundred sixty-nine individuals with chest pain who were sent to the Emergency Room were reviewed.
- Classification of the HEART score: 66 patients ranked at level 0–3 (low risk), 173 at level 4–6 (intermediate risk), and at level 7–10 (high risk)) ranked 30 patients.
- Forty-eight cases hospitalized belong to the classification 1-moderately suspicious and 21 cases hospitalized belong to the classification 2-highly suspicious classification.
- Hospitalization and troponin level had a strong positive link (HEART score), p value of 0.043.

According to estimates, 17.9 million people die from cardiovascular diseases each year, accounting for 32% of all fatalities worldwide^1^. Coronary heart disease, cerebrovascular disease, rheumatic heart disease, and other illnesses are included in the group of heart and blood vessel disorders known as cardiovascular disease. Finding individuals with acute coronary syndrome (ACS) in these patients is the first difficult task. Since the prognosis improves significantly when ACS patients receive focused treatment as soon as possible this diagnostic procedure should be quick and effective^2^. Approximately 80% of patients with chest discomfort in contemporary practice appear without a definite ACS^3^. One of the most frequent and potentially dangerous presenting complaints for adult emergency department (ED) visits is chest pain^4^. A number of different cardiac and noncardiac disorders that produce chest discomfort need to be recognized from the ACS^5^. Most hospitalized patients first visit the ED, where doctors determine whether hospitalization is necessary^6^.

Even in our country, in recent years there has been an annual increase in heart diseases. We are the first to start using HEART score, and we believe and hope that we will start using it in our daily work in the emergency clinic. These diseases in Kosovo cause more than half of the deaths, occupying the first place of all deaths, so they can be considered as the diseases with the greatest weight in our society. The prevalence of the group of cardiovascular diseases in Kosovo is 10.4% in relation to all other groups of diseases where the diagnosis of hypertension as a risk factor in the total group of cardiovascular diseases participates with 78.9%, and ischaemic heart diseases 4.9%. Ischaemic heart diseases mostly occur in men with 60%. According to age, people over 65 are more affected^7^. In the Emergency Clinic, diseases of the cardiovascular system, neurological system, pulmonary system, metabolic diseases, as well as accidental conditions dominate, without neglecting the resuscitation room where we accept the sick and injured who are in danger of life. Within 24 h in the emergency clinic, on average, more than 320 cases require medical assistance, which is a large number^8^. Cardiac emergency triage and the therapeutic decision are the most important issues in the management of ACS^9^.

One of the biggest issues facing doctors who work in emergency rooms is evaluating patients with chest pain. One emergency for chest pain is thought to be addressed for every 1000 residents served by a referral hospital, accounting for between 5 and 20% of all admissions to the emergency room^10^. The HEART score, presented in Table 1, developed by researchers^4,^ as a rapid risk stratification tool for patients with chest pain according to their short-term risk major adverse cardiac events (MACE). It is defined as acute myocardial infarction, need for percutaneous coronary intervention or coronary artery bypass graft, and death within 6 weeks. The tool helps identify low-risk patients, suitable for earlier ED discharge within 30 days of index ED visit^4^.

**Table 1:** HEART score for patients with chest pain

History (anamnesis)	
Highly suspicious	2
Moderately suspicious	1
Slightly suspicious	0
ECG	
Significant ST-deviation	2
Non-specific repolarisation disturbance	1
Normal	0
Age	
≥65 years	2
45–65 years	1
≤45 years	0
Risk factors	
≥3 risk factors or history of atherosclerotic disease	2
1 or 2 risk factors	1
No risk factors known	0
Troponin	
≥3× normal limit 3× normal limit	2
1–3× normal limit 3× normal limit	1
≤normal limit	0
	Total

ECG, electrocardiogram.

The scoring criteria for HEART are: “H” for history, “E” for electrocardiogram, “A” for age ( defined in years), “R” for risk factors, and “T” for single serum troponin, all obtained during the ED evaluation^11^. The most important behavioural risk factors of heart disease are well known and these “intermediate risks factors” can be measured in primary care facilities and indicate an increased risk of heart attack, stroke, heart failure and other complications^1^. Approximately 94% of cardiac troponin T exists as a component of the structural protein of myofibrils, and the remaining 6% is a soluble fraction in the cytoplasm^12^. Therefore, the release kinetics of troponin T reflects 1 of 2 types of myocardial injury, either the loss of the integrity of the cell membrane or progressive irreversible necrosis of myofibrils^13^. Measuring the level of troponin T is useful not only for early diagnosis of acute myocardial infarction but also for detecting high-risk unstable angina and non-Q wave infarction^14,15^.

It is crucial to consider the HEART score’s negative predictive value (NPV) while evaluating its importance in clinical practice. The NPV is a measure of the proportion of patients with a negative HEART score who do not have a MACE after 30 days. In other words, this statistic indicates the likelihood that a patient with a low HEART score is indeed at low risk for MACE. Numerous trials have revealed high NPV values for the HEART score, suggesting its effectiveness in identifying those who are unlikely to have a MACE. For instance, a research discovered that the NPV of the HEART score was 98.2% for individuals with a score of 0–3^16^.

Troponin I (Trop I) duration in relation to the time of chest pain presentation can provide important diagnostic information on the potential for ACS in individuals presenting with chest pain. When the heart muscle is damaged, the blood is created with a cardiac biomarker known as Trop I. Trop I levels that are elevated can indicate myocardial injury, which is why they are routinely utilized to diagnosis ACS. When and how much damage was done can be determined in part by the length of Trop I elevation.

The duration of the Trop I rise might vary according on the degree of cardiac infarction, with more serious injuries resulting in longer elevation durations. Trop I levels usually recover to normal 5–14 days after the onset of symptoms.

Because earlier measures might not have accurately recorded the severity of the myocardial damage, the timing of the Trop I test is critical. However, waiting too long to measure Trop I can also result in false negatives because levels may have already reached a steady state. As a result, guidelines advise measuring Trop I both at presentation and once more 3–6 h following the onset of symptoms.

The aim of the study is to exactly evaluate risk factors according to HEART score and conforming to that, to decide whether patients should be hospitalized for further evaluations and treatments.

## Methods

This study was performed at Emergency Clinic in Prishtina, Kosovo. It is a cross sectional study that included 269 patients. This population-based, study assessed ED visits through HEART score for patients with CAD and CP from January 2022 till January 2023. Inclusion criteria for this study were any patient admitted to the emergency room due to chest pain irrespective of age, prehospital assumptions, and previous medical treatments. Exclusion criteria was the age of the patients, and thus patients under age of 18 were excluded from the study. Prior to participating in the study, a verbal consent was taken from the participants as per suggestions for diagnostic procedure. This study is registered in research registry with unique identifying number researchregistry8708. Statistical analyses were done through RStudio (v2022.07.2+576). Pearson test is applied and p value less than 0.05 was considered statistically significant. The study was conducted according to STROCSS guidelines^17^.

## Results

In our study we included 269 emergency patient, 67% were male and 33% were female. Regarding the age group, 101 patients (37%) belong to the age group older than or equal to 65 years, 134 patients (50%) belong to the age group 45–65 years, and 34 patients (13%) belong to the age group younger than or equal to 45 years. Regarding risk factors, 80 patients (30%) had diabetes, 173 patients (64%) had hypertension, 67 patients (25%) had a positive family history, 59 patients (22%) were smokers, and 49 patients had hypercholesterolaemia (18%). Regarding laboratory analysis, troponin level is classified according to HEART score, so that 60 (22%) had troponin values older than or equal to 3× normal limit, while 51 cases (19%) had troponin values 1–3× normal limit and 158 cases (59%) were with troponin values≤normal limit. Of the total number of patients included in the research, 72 patients (27%) were hospitalized. As for the classification of the HEART score: 66 patients (25%) are ranked at level 0–3 (low risk), 173 (64%) are ranked at level 4–6 (intermediate risk), and at level 7–10 (high risk) ranked 30 patients (11%), as seen in Table 2.

**Table 2:** Characteristics of patients admitted to the emergency department

Characteristic	HEART care, *n* (%)
Demographic	
Male	179 (67)
Female	90 (33)
≥65 years	101 (37)
45–65 years	134 (50)
≤45 years	34 (13)
Cardiac risk factors	
Diabetes mellitus	80 (30)
Hypertension	173 (64)
Positive family history	67 (25)
Current smoking	59 (22)
Hypercholesterolaemia	49 (18)
Laboratory results at presentation	
Troponin level	
≥3× normal limit	60 (22)
1–3× normal limit	51 (19)
≤normal limit	158 (59)
Hospitalization	72 (27)
HEART score	
0–3 (low risk)	66 (25)
4–6 (intermediate risk)	173 (64)
7–10 (high risk)	30 (11)

**Figure 1:**
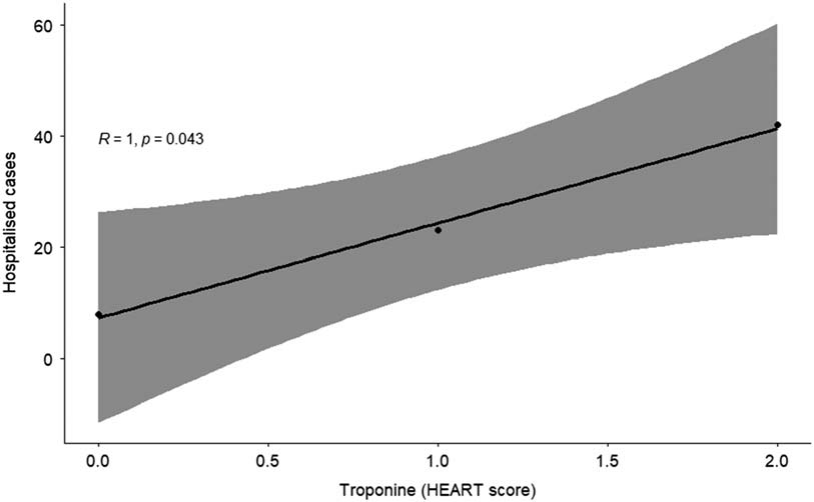
Correlation between troponin level (HEART score) and hospitalization.

In our study we aimed to find correlation between troponin level (HEART score) and hospitalization. As seen from Figure 1, R=1 indicates a strong positive correlation, also *p* value 0.043 is typically considered to be statistically significant.

**Figure 2:**
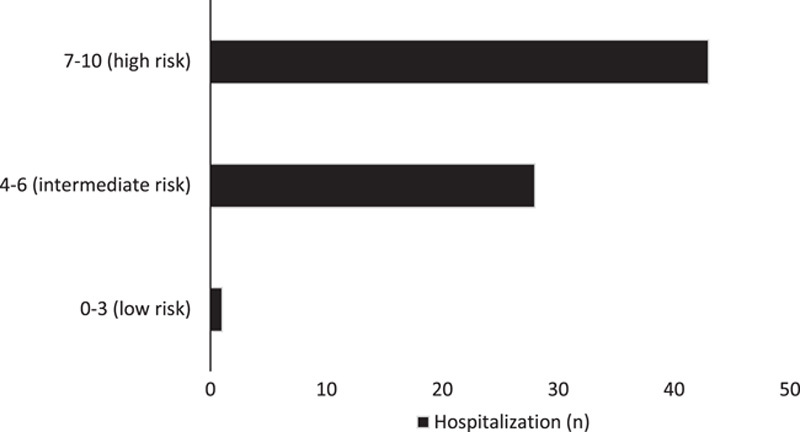
Cases classified by HEART score in relation to hospitalization.

Also, after analyzing the number of hospitalized cases with the cases classified by the HEART score, we found that from the group classified 0–3 (low risk) 1 case (1) was hospitalized, from the group 4–6 (intermediate risk) 28 (39%) cases were hospitalized, and from the group with 7–10 (high risk) 43 cases (60%) were hospitalized, presented in Figure 2.

**Figure 3:**
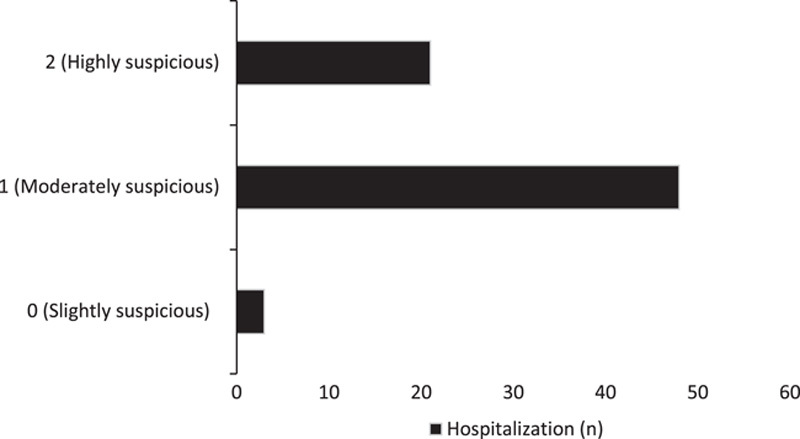
Anamnesis (positive history) in relation to hospitalization.

While after analyzing the anamnesis (history) on cardiovascular disease in relation to hospitalization, 3 cases (4%) belong to the classification 0-slightly suspicious, then 48 cases (67%) belong to the classification 1-moderately suspicious and 21 cases (29%) belong to the 2-highly suspicious classification, as shown in Figure 3.

## Discussion

According to the results of our study, around 50% of all patients who reported to an emergency room complaining of chest pain were considered to be at intermediate risk. In a situation where the medical literature is currently lacking in direction and characterized by uncertainty, the HEART score aids the cardiologist in making precise diagnostic and treatment decisions. The HEART score can be utilized for triage since it is an easy, rapid, and accurate predictor of outcome in patients with chest discomfort^4^. The HEART score has the significant benefit of facilitating doctor-to-doctor communication; one figure summarizes at least 10 lines of descriptions and concerns regarding patients with chest discomfort. For instance, when the resident calls the supervisor to discuss how to allocate scarce resources between two patients with chest pain—one with eight points and the other with two points—the options may seem obvious^4^. Additionally, one could use different variations of HEART score, which involve different protocols to further assess patients. Those variants use interpretations of various cardiac troponin assay protocols^18,19^, or additionally a hs-cTnT protocol^20^ or even another variation called HEART 0-h/1-h pathway^21^. Even though there exist variations, the HEART score is used throughout studies to support the cardiologist in emergency decisions.

The NPV of the HEART score can vary depending on the population being investigated and the pre-test likelihood of having a MACE. For instance, if the pre-test probability of MACE is higher in a given group, the NPV of the HEART score may be lower because there may be more false negatives. Therefore, when applying the HEART score and evaluating its NPV, the pre-test likelihood must be taken into account. In our situation, there was no pre-test conducted.

It is crucial to note that the NPV of the HEART score only applies to the very short-term (about 30 days) risk of MACE. Patients with low HEART scores may continue to be at risk of having cardiac episodes after this point, necessitating ongoing monitoring and risk stratification.

In addition to having a high NPV, the HEART score has additional qualities that make it appropriate for use in the ED scenario. It is a simple calculator that can be used to calculate results quickly and does not need a lot of testing or images. This might shorten the triage process and reduce the number of unnecessary tests and hospital admissions.

On top of that, in people with suspected ACS, the duration of Trop I elevation in relation to the time that chest pain initially appears can provide critical diagnostic information on the timing and amount of myocardial injury.

There were several limitations and challenges throughout the study. One of the limitations of this study is number of patients involved. Even though we tried to make the sample as much as possible representative, we are aware that we could have involved even more patients in the study.

Moreover, we have not included patients under age of 18 years old, which might be considered as selection bias. Despite different challenges, we believe that our study provides findings to conclude that HEART score can be used as a tool in our department.

## Conclusion

HEART score is a good tool which helps us in the evaluation of the patient for further treatment. The HEART score provides a quick and reliable predictor of outcomes in chest pain patients presenting to the ED. It is also useful for early and safe disposition from a busy ED without compromising patient safety. When used in conjunction with clinical gestalt and shared decision making, the HEART score can be a trustworthy tool for clinical decision making and risk classification in patients with chest pain. In our study, we can conclude that from the total sample of patients, the majority of them are associated with intermediate risk. These patients can be further observed and evaluated for further treatment, which helps colleagues to decide easier. In the future, we tend to use also different variations of the HEART score and compare the results between themselves, so that we can propose the most useful variation of HEART score.

## Ethical approval

NA.

## Consent

Prior to participating in the study, participants provided approval in the form of a verbal consent form.

## Source of funding

None.

## Author contribution

P.R., A.R.-B., and A.S.: conceptualization, data curation, formal analysis, writing—original draft. K.H., H.D., Valdet .B., Valon B., L.S., V.H., and V.L.: conceptualization, gathering data. P.R.: conceptualization, formal analysis, writing—original draft; A.S.: correspondence.

## Conflicts of interest disclosure

The authors have no conflicts of interest to declare.

## Research registration unique identifying number (UIN)

researchregistry8708.

## Guarantor

All.

## Provenance and peer review

Not commissioned, externally peer-reviewed.
